# Light intensity modulation by coccoliths of *Emiliania huxleyi* as a micro-photo-regulator

**DOI:** 10.1038/srep13577

**Published:** 2015-09-01

**Authors:** Yuri Mizukawa, Yuito Miyashita, Manami Satoh, Yoshihiro Shiraiwa, Masakazu Iwasaka

**Affiliations:** 1 Graduate School of Advanced Sciences of Matter, Hiroshima University, Hiroshima 739-8527, Japan; 2 Research Institute for Nanodevice and Bio Systems, Hiroshima University, Hiroshima 739-8527, Japan; 3 Faculty of Life and Environmental Sciences, University of Tsukuba, Tsukuba 305-8572, Japan; 4 CREST, Japan Science and Technology Agency (JST), Tsukuba 305-8572, Japan; 5 PRESTO, Japan Science and Technology Agency (JST), Saitama 332-0012, Japan

## Abstract

In this study, we present experimental evidence showing that coccoliths have light-scattering anisotropy that contributes to a possible control of solar light exposure in the ocean. Changing the angle between the incident light and an applied magnetic field causes differences in the light-scattering intensities of a suspension of coccoliths isolated from *Emiliania huxleyi*. The magnetic field effect is induced by the diamagnetic torque force directing the coccolith radial plane perpendicular to the applied magnetic fields at 400 to 500 mT. The developed technique reveals the light-scattering anisotropies in the 3-μm-diameter floating coccoliths by orienting themselves in response to the magnetic fields. The detached coccolith scatters radially the light incident to its radial plane. The experimental results on magnetically oriented coccoliths show that an individual coccolith has a specific direction of light scattering, although the possible physiological effect of the coccolith remains for further study, focusing on the light-scattering anisotropies of coccoliths on living cells.

Microorganisms have tremendously affected the Earth’s environment^1^,^2^,^3^,^4^. In particular, some species of coccolithophore phytoplankton have played a significant role in carbon fixation, which resulted in the accumulation of fossil fuels and the generation of the gas atmosphere that supports all living organisms^5^,^6^,^7^,^8^,^9^,^10^,^11^.

Moreover, other species of coccolithophores produce crystals of calcium carbonate^5^,^6^,^7^,^8^,^9^,^10^,^11^,^12^,^13^,^14^,^15^,^16^,^17^,^18^,^19^,^20^,^21^,^22^,^23^,^24^,^25^,^26^,^27^,^28^, such as calcareous cell coverings, via biomineralization within the Golgi vesicles inside their cells^18^,^19^,^20^,^21^. For example, the calcite crystals of *Emiliania huxleyi* form a complicated structure that combines components with different crystallographic orientations; this formation is known as the V/R nucleation model^19^. Large blooms of coccolithophores promoted the accumulation of coccoliths and thus the formation of the famed White Cliffs of Dover during geological events, especially during the Cretaceous Era.

Although the cell surface location of coccolith disks may provide adaptability to sun exposure in the ocean, the detailed mechanisms and explanations of their functions are incomplete. Additionally, many other aquatic organisms possess biomineralization mechanisms for producing biogenic crystals, including calcite and aragonite, in cell coverings, shells, and body parts. Previous studies have suggested that these types of biogenic inorganic crystals serve as optical regulators, in addition to being skeletal elements. For example, the dome-shaped calcite on the surface of the brittle star functions as an optical lens^29^,^30^. The coccoliths produced by coccolithophores are designed on a nanometer scale, whereas the calcites of the brittle star and sea urchin are designed on a micrometer scale. Currently, the nanostructures of coccoliths are too complicated to be artificially replicated by modern technology.

The most important question concerning coccolith function is how they manage solar light exposure in the ocean. Coccolithophore blooms appear as a color difference in ocean satellite images, and the light scattering properties of coccoliths are believed to be the cause of this phenomenon. Recently, studies that focus on the optical function of coccoliths have been reported. For example, one study revealed that a holococcolith functioned as an optical filter for ultraviolet light^22^. The light that is incident on the coccoliths was highly scattered, and the cells with coccoliths scattered the incident light more efficiently^23^,^24^. In a previous study, the researchers measured the light scattering of randomly oriented coccoliths. In our study, the magnetic orientation of coccoliths enables us to investigate the direction of light scattering from coccoliths. The idea that light may be diffracted into the cell has been disputed because the refractive index of calcite is higher than the refractive index of water^25^. The hypotheses of these studies were that coccoliths shade the cell from strong light by scattering the light^18^ and that light diffraction and concentration may occur due to the difference between the refractive indexes of calcite and water^25^.

There are reports showing a statement that coccoliths does not improve the physiological performance of coccolithophores^26^,^27^,^28^. This suggestion is based on data showing no difference between calcified and naked cells in their photosynthetic rate in saturating light intensity^26^,^27^ and an absence of photoinhibition under strong light exposure up to 1000 μmol/m^2^s 28.

However, for a more detailed understanding of the physiological functions of coccoliths in the coccolithophore, further investigation is required on the optical properties of coccoliths detached from an Emiliania cell. The previous studies reported on the optical properties of randomly orienting coccoliths^23^,^24^, but there have been no reports that have provided experimental data on the light-scattering properties of oriented coccoliths. Therefore, a new method to determine the directional properties of light-scattering in coccoliths is needed.

Our previous study revealed that the structural color of coccoliths were affected by strong magnetic fields of more than 1 T, but the detailed mechanism was remained^31^. In this study, we examine the light-scattering properties of floating coccoliths by controlling their orientation by switching magnetic fields on/off. Analysis of the optical properties of an individual coccolith on a cell is difficult because coccoliths adhere to the cell surface and build upon one another. Therefore, we prepared an aqueous suspension of isolated coccoliths from *Emiliania* cells and observed their light-scattering behavior in static magnetic fields which can control the orientation of coccoliths. Using such novel approach developed in this study, the light-scattering functions of floating coccoliths can be evaluated.

## Results

### Diamagnetic orientation of coccoliths

**Figure 1:**
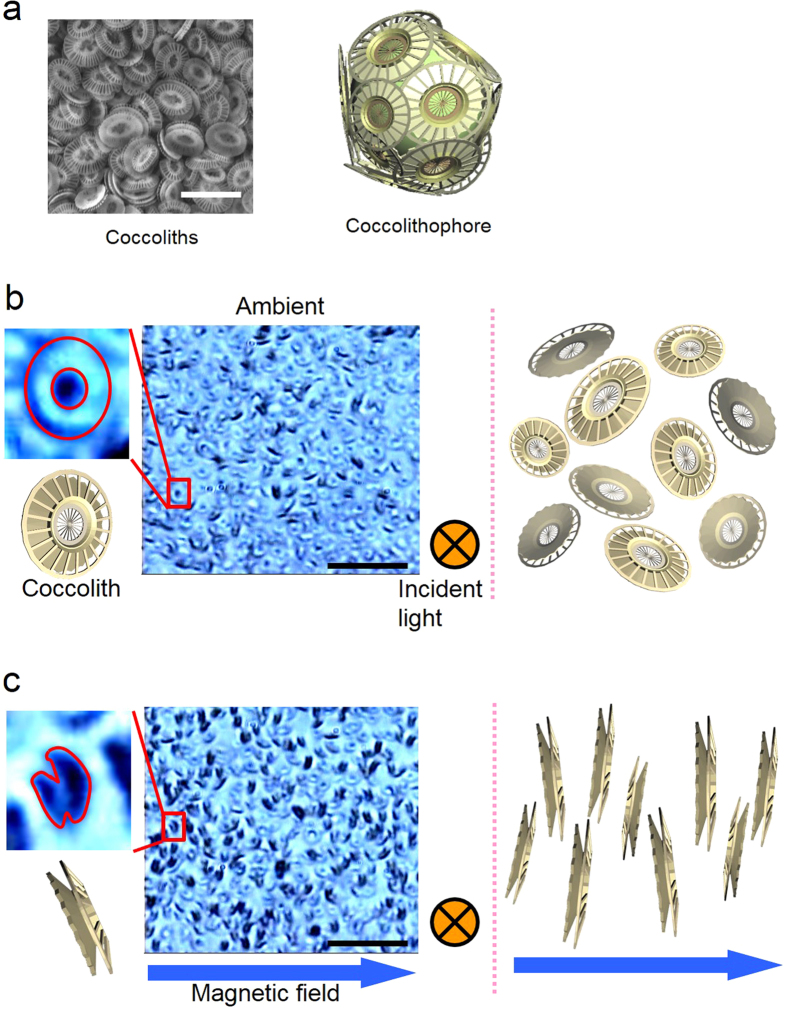
Magnetic orientation of coccoliths. (**a**) SEM image of isolated coccoliths of *Emiliania huxleyi* and a model *Emiliania huxleyi*. Bar, 5 μm. (**b**) Bright field image of coccoliths in the absence of a magnetic field. Bar, 20 μm. (**c**) Changes in the inclination of coccoliths subjected a magnetic field of 400 mT (left-right direction).

Figure 1 shows a change in the inclination of coccoliths in a magnetic field of 400 mT. In the photograph (Fig. 1c), the observed shapes of coccoliths suggest an increase in the number of coccoliths in which the direction of the radial board is perpendicular to the applied magnetic field, whereas most of the coccoliths shown in Fig. 1b are randomly oriented. The coccoliths with a diameter of 3 μm demonstrated Brownian motion when floating in water, and the thermal fluctuation at room temperature caused disorder in the coccolith orientation. However, the percent of coccoliths oriented to the same direction increased during the magnetic field exposure.

**Figure 2:**
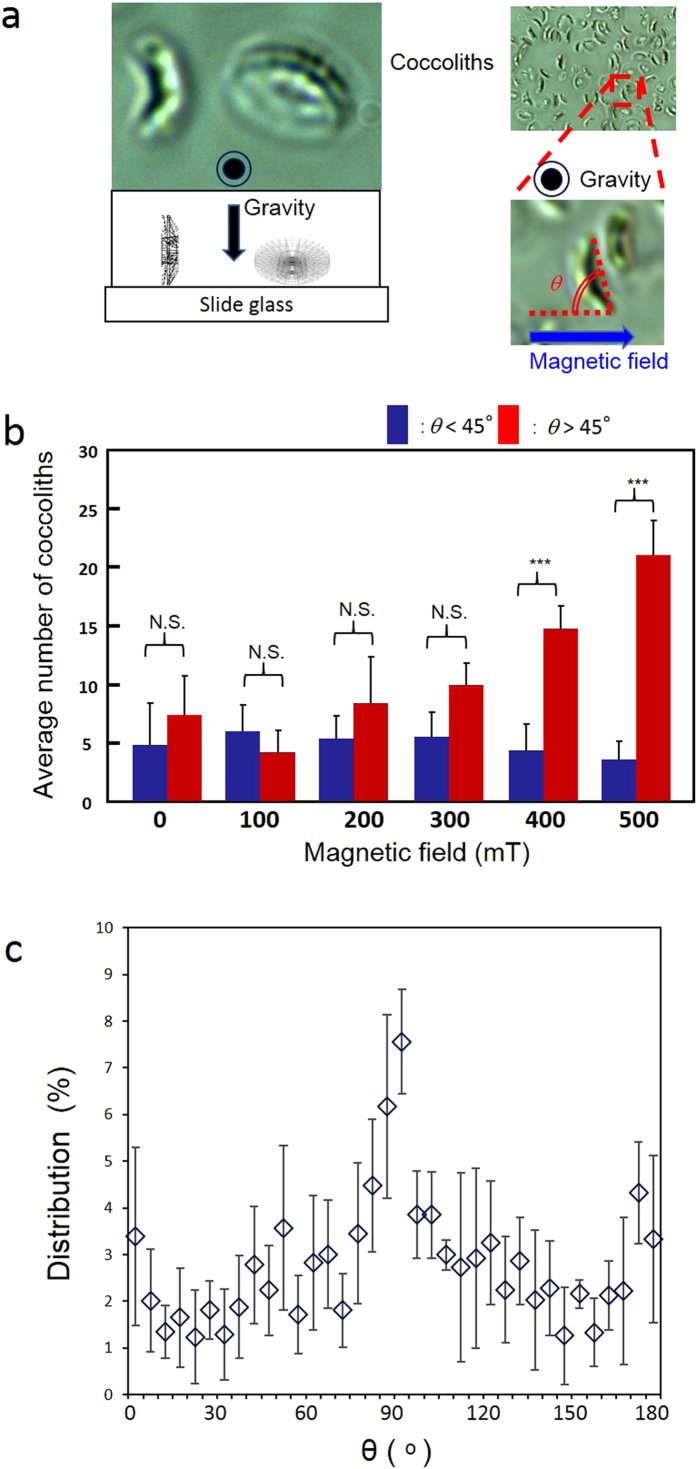
Statistical analysis of the angles of diamagnetically oriented coccoliths. (**a**) Definition of the orientation angle θ. (**b**) Average number of gravity-parallel coccoliths per analyzed image; the coccoliths were categorized as having θ less than and greater than 45° (N = 5). (**c**) Averaged distributions of θ of coccoliths exposed to a 500 mT magnetic field. Distributions were plotted from 0° to 180°.

Figure 2 shows the statistical analysis of the oriented coccoliths. In the early process of the magnetic orientation, the number of coccoliths that directed their radial board parallel to Earth’s gravitational field increased (Fig. 2a). We evaluated the angle of the applied magnetic fields with the radial direction, and we determined how the change in orientation depended on the magnetic fields (Fig. 2b). The number of coccoliths in the same direction did not differ for magnetic fields of 0 mT to 300 mT. However, the number of coccoliths that were oriented perpendicularly to the 400 mT to 500 mT magnetic fields significantly increased. As shown in Fig. 2c, the angular distribution of the gravity-parallel coccoliths in 500 mT magnetic fields provided evidence of magnetic orientation.

### Shape and size characteristics of coccoliths, which contain several types of CaCO_3_ (calcite) crystals

**Figure 3:**
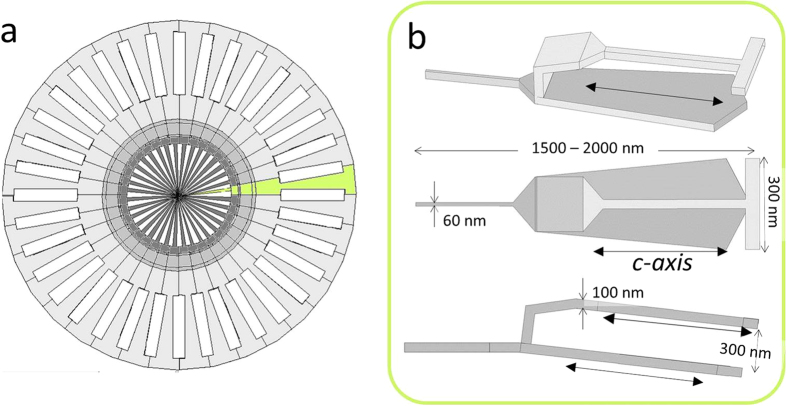
Characteristics of the shape and size of a coccolith, which consists of several CaCO_3_ (calcite) crystals. (**a**) Approximated model of the coccolith of *Emiliania huxleyi* (over view). (**b**) Unit of calcite crystals. These units are radially combined to create a disk of the coccolith. The c-axis of calcite crystals is parallel to the radial direction.

The coccoliths that were used in this study were obtained from *Emiliania huxleyi*. Figure 3 displays the redrawn images of a coccolith and its unit by J. R. Young and K. Henriksen^32^. A coccolith consists of the units shown in Fig. 3b that are composed of calcite crystals. The approximate sizes of the unit in Fig. 3b were determined by referring to the SEM image. According to previous studies^32^,^33^, the c-axis of the calcite crystal of an *E. huxleyi* coccolith is directed parallel to the radius of the disk.

This study suggested that the c-axis of the coccolith units prefers to be directed perpendicular to the magnetic fields. The coccoliths do not contain strongly magnetic molecules because calcite is diamagnetic. However, the diamagnetic anisotropy of calcite is reported to be 4.09 × 10^−6^ (emu/g)^34^, which is sufficient to obtain a diamagnetic anisotropy energy that exceeds the thermal energy, kT.

### Light scattering of coccoliths with and without magnetic field exposure

**Figure 4:**
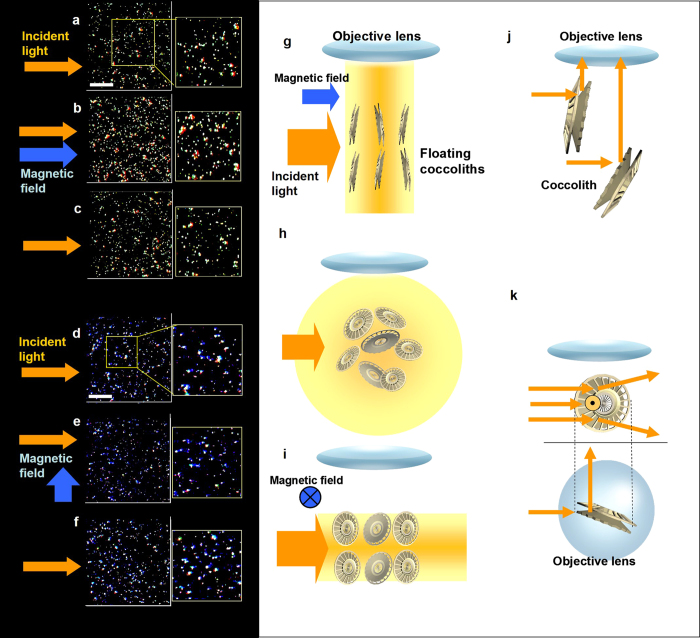
Light scattering changes in floating coccoliths in water with and without magnetic field exposure at 400 mT. An enhancement (**a**)–(**c**) and an inhibition (**d**)–(**f**) of light scattering were obtained when the incident light was parallel and perpendicular to the magnetic field, respectively. (**a**) Dark field image of coccoliths immediately before the magnetic field exposure, as well as an expanded image. Bar, 50 μm. (**b**) Enhancement of light scattering by a 400 mT magnetic field parallel to the dark field illumination. (**c**) Post-exposure images of coccoliths scattering light. (**d**) Pre-exposure image. Bar, 50 μm. (**e**) Inhibition of light scattering during the application of magnetic fields parallel to the incident light. (**f**) Post-exposure image. (**g**) Speculation and proposed model of the enhancement of light scattering in coccoliths floating in water when the coccolith orientation was affected by the diamagnetic torque force. The magnetic orientation of the coccoliths caused their radial plane to align perpendicular to the magnetic field. Consequently, the light scattered toward the objective lens became intense. The light scattering pattern (column in orange-yellow) become anisotropic and directed to the objective lens because the incident light faced the coccolith plate. (**h**) Isotropic light scattering in randomly oriented floating coccoliths. (**i**) A magnetic field orthogonal to the incident light caused the direction of the coccolith plates to be parallel to the angle of incidence of the light, and the light passed through the coccolith plates. Details of the mechanism are shown (**j**)–(**k**). (**j**) Radial plane of the backboard-facing incident light. The light is reflected to the side, and the increase in brightness was measured. (**k**) Incident light nearly parallel to the radial plane can be reflected by the curved surface in front or in back of the calcite board. In both cases, light scattering enhancements in the vertical direction can be detected when a coccolith is observed from the front or the back. In contrast, less light is scattered in the direction that is orthogonal to both the incident light and the magnetic field.

In Fig. 4a, illumination from the side caused dynamic light scattering in the direction of observation. When exposed to a 400 mT magnetic field that was parallel to the incident light, the light scattering from the coccoliths was enhanced, as shown in Fig. 4b. The diamagnetic energy can modulate coccolith rotation due to Brownian motion, which produces a change in the light-scattering intensity. The effect was reversible and reproducible. After the magnetic field was switched off, the intensity returned to the level observed prior to the exposure. In the model shown in Fig. 4g, we proposed that the radial component in the coccolith was directed perpendicular to the applied magnetic field. Consequently, we speculated that the incident light was bent by the edge of the coccolith board (Fig. 4j) and scattered to the sides.

To test this assumption, we performed the same experiment but changed the direction of incident light from a parallel to a perpendicular orientation to the magnetic field. This second condition resulted in the opposite response (Fig. 4d–f). According to our model of light control in coccoliths (Fig. 4i–k), the light that travels parallel to the radial direction passes through (Fig. 4i) or can be partially scattered in the vertical direction of the radial board of coccoliths (Fig. 4k). This modulation reduced the light scattering that was observed from a direction perpendicular to the magnetic field and the incident light.

Figures 4i–k summarize the dependence of light scattering on the orientation of the coccoliths relative to the incident light, thus providing an interpretation of the obtained light scattering behavior. The interpretation suggests that the average direction of the coccoliths is restricted by the diamagnetic anisotropy energy in calcite during their Brownian motion.

### Light scattering anisotropy

**Figure 5:**
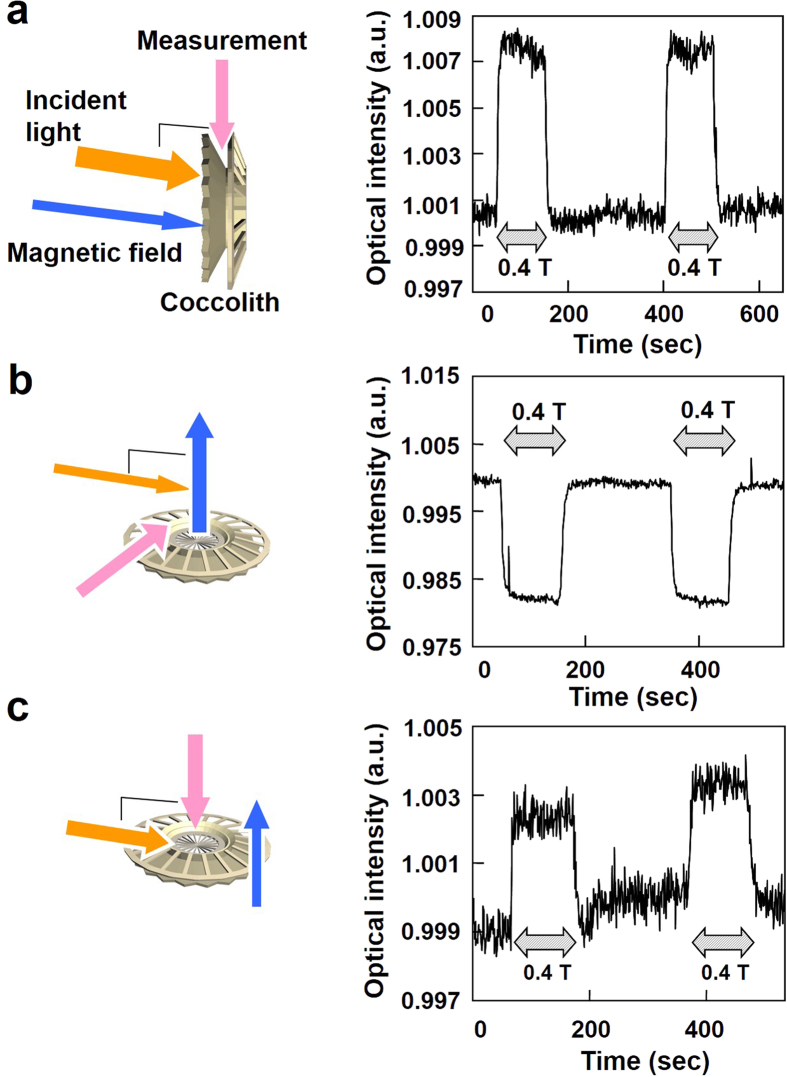
Fiberoptic detection of the light scattering intensity in the floating coccoliths when the average direction of the radial board orientation was affected by the applied magnetic field. A magnetic field of 400 mT was applied twice during the time-course measurement. (**a**) The incident light was parallel to the magnetic field. Light was collected from the direction that was perpendicular to both the incident light and the magnetic field. (**b**) The three vectors were orthogonal. (**c**) Light was collected from a direction that was parallel to the magnetic field. The incident light was perpendicular to both the detection direction and the magnetic field direction.

By employing a fiber optic measurement, we obtained the three modes of light-scattering by coccoliths in magnetic fields (Fig. 5). To investigate the light-scattering anisotropy in coccoliths, the angles of three vectors, the incident illumination, the applied magnetic field, and the detecting axis, were varied. The three directional configurations of light-scattering patterns in Fig. 5a–c were modified and are described in a time-series graph.

The first mode, in which coccoliths are oriented perpendicular to both the magnetic field and the incident light, distinctly enhanced the light scattering (Fig. 5a). The relative changes (Δ%) in light scattering intensity were increases of 0.8%. Conversely, considerable inhibition (approximately −1.8%) of the light scattering occurred when the three vectors were orthogonal to one another (Fig. 5b). The light-scattering parallel to the applied magnetic field in dark-field illumination (the third mode, Fig. 5c) exhibited an increase of 0.35% in light intensity. The time series of the changes in the scattered light intensity support a distinct relationship between light scattering and the directional configurations of the three vectors: (magnetic field, incident light, and observation).

## Discussion

From these results, we can conclude that the coccolith disks on *Emiliania huxleyi* perform roles in reducing and enhancing the light that enters the cell by scattering the light. The light-scattering intensities at the angle of 90 degree, which are shown in Fig. 5, indicate increase and decrease in the light-scattering occurs depending on the angle of incident light inserted into the coccoliths.

We conclude that the individual coccolith disc has the ability to efficiently control the incident light, although it is still unclear whether coccoliths attached on the cell surface exhibit light shades or light collectors for the cell inside when they form a coccosphere.

This new method enables the quantification of the light-scattering properties of free coccoliths with a given orientation. Utilization of this method may also enable a better approximation of the effect of coccoliths on the intracellular light environment and provide insight into other optical functions such as polarization of light, light protection and light amplification. The present study does not provide any evolutionary properties of coccoliths with respect to light scattering. A precise evaluation of the coccoliths’ light-scattering anisotropy can be performed through experiments on intact coccolith-bearing cells, studying the photo-inhibitory response under strong incident light.

Currently, no artificial method exists to reproduce precise structures, such as a coccolith without a coccolithophore. Although *in vitro* calcification can be achieved by utilizing an organic template that can be extracted from cells, the resulting crystallized calcite is larger than the coccoliths from coccolithophores^11^. The methods developed in this study enabled us to observe the light-scattering anisotropy in a group of coccoliths by controlling their orientation via their diamagnetic orientation. We can expect other optical functions of coccolith discs. For example, accumulated coccolith discs can generate a photonic crystal property which will control the light propagation. In the future, we intend to clarify the light-controlling properties of individual coccoliths and their components using a more precise optical measurement system. Furthermore, the ability of coccoliths to modify light suggests their potential in applications as new micro/nano optical devices.

## Methods

### The preparation of coccoliths from microalgae cells

*Emiliania huxleyi* (Lohmann) Hay & Mohler (B349) was obtained from the Plymouth Culture Collection of Marine Microalgae, currently in Marine Biological Association of the UK, by the help of Dr. K. Hagino (Kochi University, Kochi, Japan). The algal cells were grown at 20 °C in a 500 mL-glass vessel containing the artificial seawater medium (Marine Art SF containing ESM microelement enrichment in which soil extract is replaced with 10 nM sodium selenite)^35^. The culture suspension was maintained under continuous illumination by fluorescent lamps at the intensity of 100 μmol/m^2^/s and bubbling with air at 100 mL/min. The coccoliths were collected using a centrifuge at 3500 rpm (3000 × g) for 10 min, and the obtained precipitate was mixed with 0.25% trypsin after removing the supernatant.

### Magnetic field generator

A resistive electromagnet (Hayama Co. Ltd., Japan) with a maximum intensity of 500 mT was used in this study. The region of magnetic field exposure was between the two magnet poles (100 mm in diameter). The gap between the poles was adjusted from 130 mm to 150 mm. A sample chamber that contained the coccoliths was placed in this space in front of the lens of a CCD microscope (with the lens in the exposure space). The sweep rate of the magnetic field in the resistive magnet was 400 mT/sec.

### Microscopy observations

We used two types of CCD microscopes for observation, a high-amplification microscope (Keyence, VH-5000 with VHS501 lens, 1000 × magnification, United States) and a low-amplification microscope (ELMO, CC421, United States). In the experiments with the high-amplification microscope, the chamber (flame seal chamber, BIO-RAD SLF-0201, United States) had a 25-μl capacity that could hold the coccoliths, which were observed through a glass plate. The collected coccoliths were contained in a closed chamber of thin glass, which was placed statically and horizontally on the CCD camera head.

### Fiber-optic spectroscopy under applied magnetic fields

Previous studies on the light scattering properties of coccoliths were carried out by utilizing a backscattering measurement system, which provided information about the light scattering properties of randomly oriented coccoliths floating in aqueous medium. In this study, we constructed an optical measurement system for backscattering at an angle of 90 degrees (scattering to side) between the magnetic poles of an electromagnet. The apparatus exhibited the evaluation of the light-scattering intensity on a radial plane and the edge of the coccoliths macroscopically because the magnetic fields controlled the direction of the coccoliths relative to the incident light. The orientation of the coccoliths was confirmed by the abovementioned microscopy observation system with the electromagnet.

The fiber-optic measurements were conducted with three configurations of sample units, which were positioned between the magnetic poles. The sample cuvette was illuminated using a halogen lamp (Focusline, Philips Co., Ltd., the Netherlands). An optical spectrometer (Shamrock: SR-303i-A, Andor, Co. Ltd. United Kingdom) and cooling CCD sensor (iDUS: DV420A-OE, Andor, Co. Ltd. United Kingdom) were used as optical detectors.

The optical measurements and analyses were performed as follows. The scattered light intensities at wavelengths at 500 nm to 600 nm measured continuously. The magnetic field exposure was initiated after the light intensity reached a steady level. After the data acquisition, off-line analyses were conducted. For the first analysis, the light intensities were normalized using the steady value of the initial level before the first exposure, which was set as 100%. Then, the relative change (Δ%) was obtained by subtracting the initial level (100%) from the normalized value (%).

### Statistical analysis of the angles of diamagnetically oriented coccoliths

The number of magnetically oriented coccoliths was statistically analyzed using the orientation angle θ, which was defined as the angle between the applied magnetic field and the radial board of the coccolith directed parallel to Earth’s gravitational field. For one set of analyses, a photograph of a 29 μm × 22 μm area was utilized. Within this area, 10 to 20 coccoliths directed their radial plane parallel to Earth’s gravitational field when the sample was not exposed to the magnetic fields, and the number of the gravity-parallel coccoliths increased when the magnetic field exposure of 400 mT to 500 mT was initiated. As shown in the right panel of Fig. 2a, by selecting the gravity-parallel coccoliths, we can measure the orientation angle θ. In the analysis shown in Fig. 2b, the numbers of coccoliths with θ less than and greater than 45° in magnetic fields of 0, 100, 200, 300, 400, and 500 mT were determined. The experimental number was defined as the number of images that were utilized to analyze θ.

## Additional Information

**How to cite this article**: Mizukawa, Y. *et al.* Light intensity modulation by coccoliths of *Emiliania huxleyi* as a micro-photo-regulator. *Sci. Rep.* **5**, 13577; doi: 10.1038/srep13577 (2015).
